# Metastasis of oncocytic thyroid carcinoma in the mandibular condyle: An exceptional localization

**DOI:** 10.4317/jced.62635

**Published:** 2025-05-01

**Authors:** Noemí Vieira-Sebe, Fernando Almeida-Parra, Álvaro Ranz-Colio, Esther Moreno-Moreno, Julio Acero-Sanz

**Affiliations:** 1 Department of Oral and Maxillofacial Surgery, Ramón y Cajal University Hospital, Ramón y Cajal Health Research Institute (IRYCIS), Madrid, Spain; 2 Department of Oral and Maxillofacial Surgery, Puerta de Hierro Majadahonda University Hospital, Ramón y Cajal Health Research Institute (IRYCIS), Madrid, Spain; 3 Department of Pathology, Ramón y Cajal University Hospital, Ramón y Cajal Health Research Institute (IRYCIS), Madrid; 4 Faculty of Medicine, Alcalá University, Alcalá de Henares, Spain

## Abstract

**Introduction:**

Oncocytic thyroid carcinoma, previously known as Hürthle cell carcinoma, is a well-differentiated neoplasm accounting for 3-5% of malignant thyroid tumors. This type of carcinoma exhibits aggressive behavior with a propensity for lymphovascular invasion and distant metastases.

**Objective:**

To present an unusual clinical case of oncocytic thyroid carcinoma metastasis to the right mandibular condyle, highlighting its surgical management and clinical significance.

**Case report:**

An 86-year-old woman with a history of oncocytic thyroid carcinoma treated with total thyroidectomy and radioactive iodine in 2019. During follow-up, elevated thyroglobulin levels were detected, and a PET-CT scan revealed uptake in the right mandibular condyle. The lesion was confirmed by magnetic resonance imaging and surgical biopsy and was treated with a right condylectomy. Histopathological analysis revealed bone infiltration by oncocytic carcinoma. The patient had a favorable postoperative course, with undetectable serum thyroglobulin levels after surgery.

**Discussion:**

Bone metastases from oncocytic carcinoma are rare, with the lungs being the most common metastatic site. To our knowledge, this is the second reported case of mandibular metastasis and the first involving the mandibular condyle, underscoring the importance of radical surgical intervention and a multidisciplinary approach in such cases.

**Conclusions:**

This case highlights the aggressive nature of oncocytic thyroid carcinoma and the importance of early diagnosis and personalized treatment to improve the prognosis of bone metastases.

** Key words:**Oncocytic thyroid carcinoma, bone metastases, mandible metastases.

## Introduction

Bone metastases from oncocytic thyroid carcinoma are uncommon, and those located in the mandible are extremely rare, representing a diagnostic and therapeutic challenge that is scarcely described in the literature. Oncocytic thyroid carcinoma, previously known as Hürthle cell carcinoma, is a well-differentiated malignant neoplasm derived from follicular cells, accounting for approximately 3-5% of malignant thyroid tumors (1). This tumor exhibits more aggressive behavior than other well-differentiated thyroid carcinomas, with a higher tendency for lymphovascular invasion and metastasis to regional lymph nodes and distant organs, primarily the lungs and bones (2).

Metastases pose not only a diagnostic challenge due to their atypical clinical presentation but also a therapeutic challenge, as published evidence is limited and standardized recommendations are lacking.

We present the case of an 86-year-old patient diagnosed at our center with a metastatic lesion in the right mandibular condyle secondary to oncocytic thyroid carcinoma, highlighting the surgical management and its clinical relevance. This case aims to contribute to the existing literature and emphasize the importance of a multidisciplinary approach in the treatment of these rare lesions.

## Case Report

**Figure 1:**
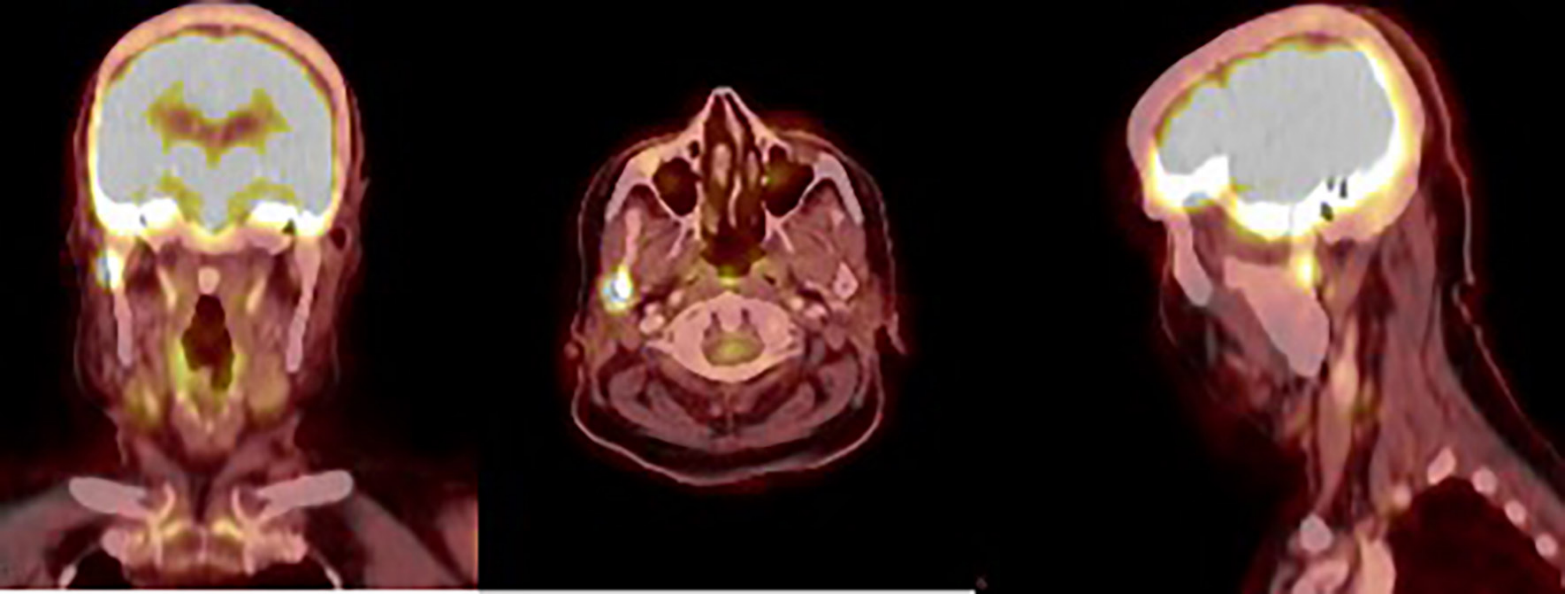
PET-CT 18F-FDG showing focal uptake in the right mandibular condyle.

An 86-year-old female patient with no relevant medical history was under follow-up by the Endocrinology Department after being diagnosed with oncocytic variant thyroid carcinoma, classified as pT3a (m) N0 M0 (stage II), in November 2019. She was treated with total thyroidectomy followed by 100 mCi of radioactive iodine (RAI). During follow-up, persistent elevated serum thyroglobulin levels were detected, prompting a 18F-FDG PET-CT scan, which revealed a focal uptake in the right mandibular condyle with a maximum SUV of 8 (Fig. 1).

**Figure 2:**
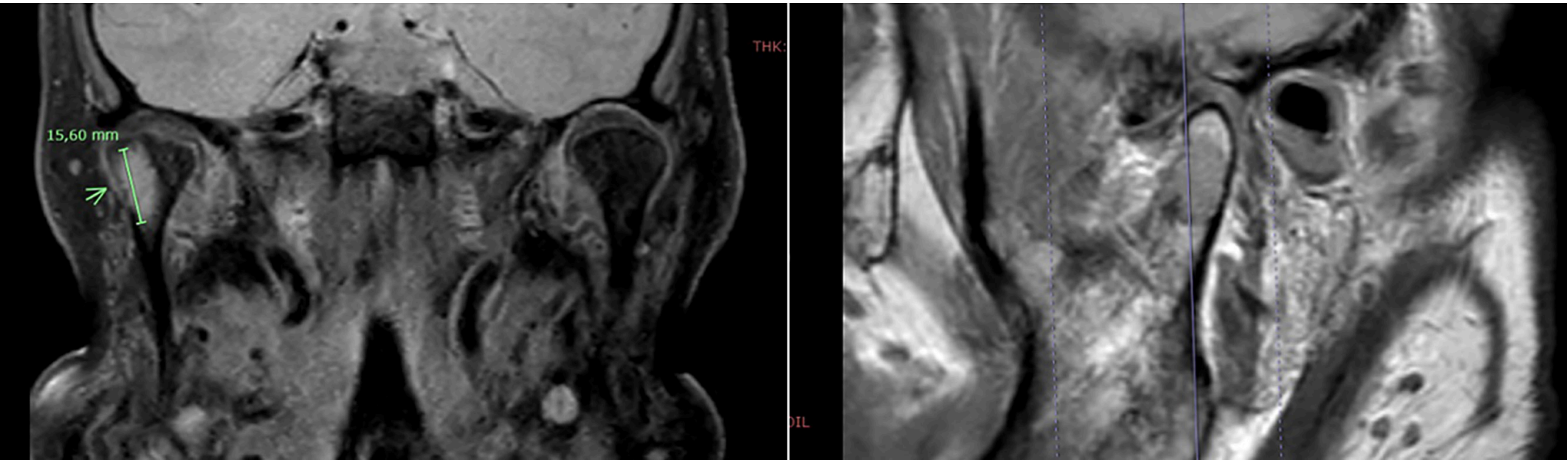
Coronal (right) and sagittal (left) MRI slices showing an eccentric lesion in the right mandibular condyle.

Further assessment with magnetic resonance imaging (MRI) confirmed the presence of a 15-mm lateral eccentric lesion in the right mandibular condyle, infiltrating the cortical bone and causing soft tissue edema, including involvement of the lateral pterygoid muscle (Fig. 2). Given the suspicion of distant metastasis, the patient was referred to our department for evaluation and management of the lesion.

**Figure 3:**
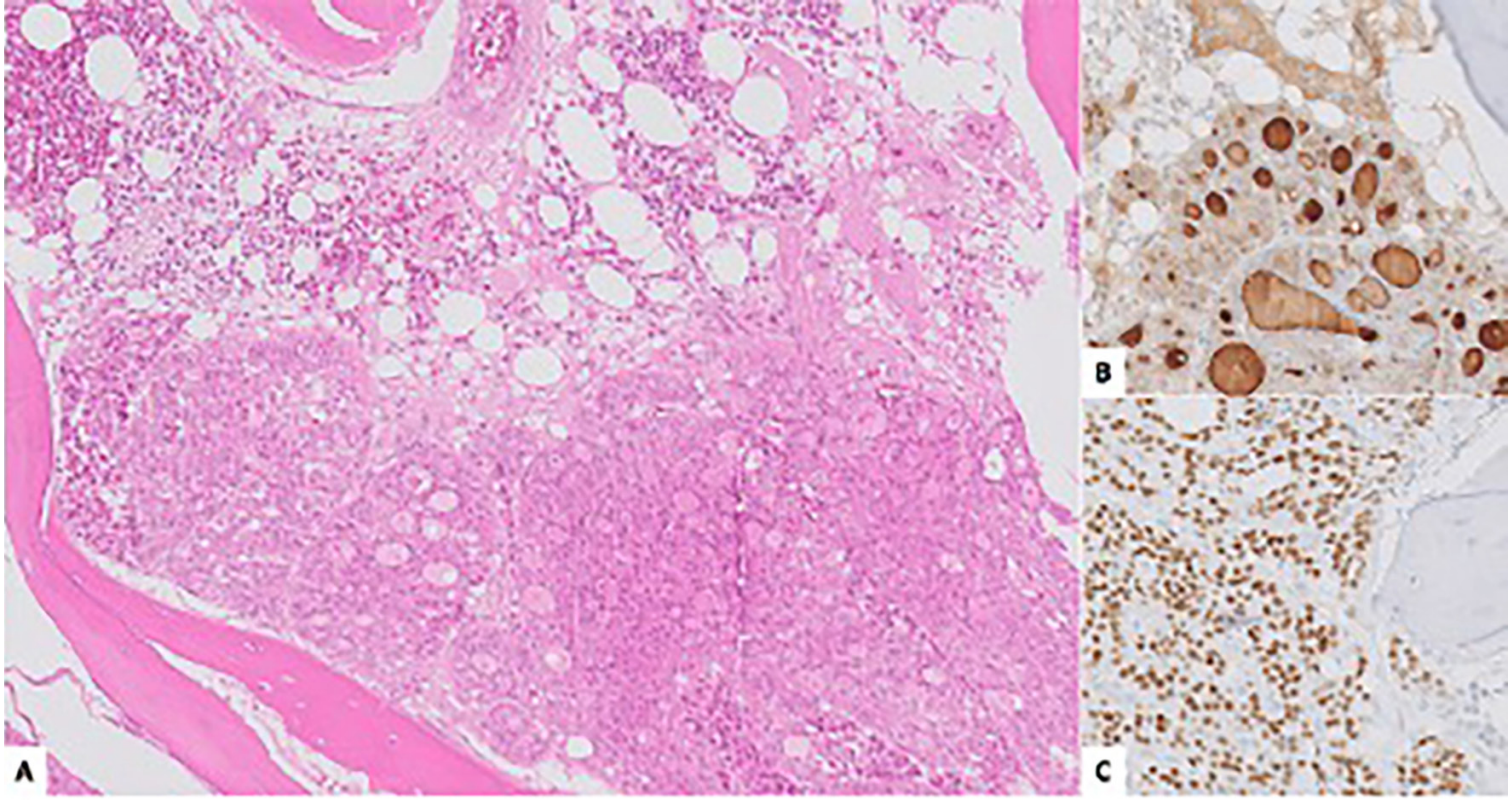
A: Bone fragment infiltrated by a neoplastic proliferation with a follicular pattern and oncocytic cell morphology (hematoxylin-eosin). Positivity for thyroglobulin (B) and TTF1 (C) is observed.

After discussion in the multidisciplinary head and neck tumor board, surgical resection of the lesion was recommended. A preauricular retroparotid approach was performed, allowing for a right condylectomy using piezoelectric surgery, with the joint capsule included as the resection margin. The surgical specimen was sent for histopathological examination (Fig. 3).

Histological analysis revealed bone infiltration by a neoplastic proliferation with a follicular growth pattern and oncocytic cytological features. Immunohistochemical analysis showed positive staining for PAX8, TTF1, and thyroglobulin, while calcitonin was negative. The proliferation index (Ki-67) was <1%. These findings confirmed the diagnosis of metastatic oncocytic thyroid carcinoma.

The patient had a favorable postoperative course and was discharged 24 hours after surgery. As a sequela, she presented with deviation of mouth opening toward the right side, without functional impairment. Immediate postoperative serum thyroglobulin levels became undetecTable, demonstrating the effectiveness of radical surgical treatment. During a 12-month follow-up period, no signs of recurrence were observed in imaging or laboratory tests.

## Discussion

Oncocytic thyroid carcinoma is considered a well-differentiated malignant tumor originating from oncocytic (Hürthle) cells, which in turn derive from the thyroid follicular epithelium. It was first described by Hürthle in 1984. Before the 2022 update of the World Health Organization (WHO) Classification of Thyroid Tumors, oncocytic thyroid carcinoma was referred to as Hürthle cell carcinoma and classified as a subtype of follicular thyroid carcinoma. Due to its distinct clinical behavior, histopathological characteristics, and molecular alterations, it has now been recognized as a separate entity (3).

This malignancy is rare, accounting for approximately 3-5% of all thyroid cancers (1,4,5). It is most frequently diagnosed in individuals over 50 years old and has a female predominance, although to a lesser extent than other thyroid malignancies (4). As with other types of thyroid cancer, several risk factors have been identified, including childhood exposure to ionizing radiation, a personal history of thyroid diseases, iodine deficiency, and exposure to endocrine disruptors and environmental pollutants (6,7).

Patients with this tumor are often asymptomatic at diagnosis. Nevertheless, oncocytic thyroid carcinoma tends to present at an advanced stage and has a worse prognosis and lower survival rates compared to other well-differentiated thyroid carcinomas (4,5,9). Goffredo *et al*. (4) compared the clinical behavior and survival of 3,311 patients with oncocytic thyroid carcinoma versus 59,235 patients with other thyroid carcinomas, demonstrating that the former group not only had larger tumors at diagnosis but also presented with more advanced disease (localized disease in 51.4% of oncocytic carcinoma cases vs. 64.3% in other thyroid carcinomas). Overall survival and disease-specific survival were lower in the oncocytic carcinoma group (82.1% vs. 89.2%). Humphreys *et al*. (10) reported a 10-year overall survival rate of 78.1% in a cohort of 4,643 patients.

These differences are attributed to the tumor’s tendency to invade lymphatic and vascular structures, leading to regional metastasis in cervical lymph nodes and distant metastases in up to 5% of cases. The most frequently affected distant sites are the lungs and bones (5,11,12-14).

Regarding the primary diagnosis of this tumor, according to the American Thyroid Association guidelines for managing thyroid nodules, imaging and histopathological examination of the surgical specimen are essential. Ultrasound, including evaluation of both the central and lateral neck compartments, is the imaging modality of choice. CT and MRI are reserved for cases with suspected advanced disease and lymph node involvement. Based on ultrasound findings, a biopsy may be indicated, with fine-needle aspiration biopsy being the recommended approach (15). The definitive diagnosis of oncocytic thyroid carcinoma is confirmed through histopathological analysis demonstrating vascular or capsular invasion in the examined specimen (8).

Molecular characterization has identified thyroid carcinoma subtypes with different clinical behaviors and varying susceptibility to radioactive iodine and targeted therapies. In patients with metastatic disease unresponsive to radioiodine therapy, molecular characterization can identify mutations that may be targeted with specific treatments. The most common genetic alterations driving oncocytic carcinoma growth involve proteins in the mitogen-activated protein kinase (MAPK) pathway (1).

The optimal treatment strategy for this carcinoma remains controversial due to the lack of prospective studies validating the extent of thyroidectomy and the use of radioactive iodine (RAI) adjuvant therapy. According to the American Thyroid Association and the National Comprehensive Cancer Network guidelines, total thyroidectomy is the preferred therapeutic approach, similar to other well-differentiated thyroid carcinomas. If lymph node involvement is present, central (level VI) and lateral neck dissection of the affected compartments should be performed (2,8,15). Adjuvant RAI therapy is less effective for oncocytic carcinoma than for other histological types, as only 10% of cases demonstrate iodine uptake (5,6). The role of external beam radiation therapy (EBRT) and chemotherapy remains uncertain for this type of tumor (8).

The literature on the management of oncocytic carcinoma metastases is limited. In general, available treatment options include surgical resection, when feasible given tumor location and patient status, as well as radioiodine ablation, external beam radiation therapy, chemotherapy, tyrosine kinase inhibitors, or a combination of these approaches (12-14,16). Both radiation therapy and chemotherapy have been described for treating locoregional recurrences and as palliative options for symptomatic disease (5,12-14,16).

In a series of 32 patients with oncocytic carcinoma metastases published by Besic *et al*. (12,13) patients received external beam radiation therapy for metastatic lesions with effective results. Among the 13 patients with distant metastases, 9 had bone involvement, with an observed symptomatic palliative effect lasting an average of 93 months. In another study by the same author, 16 patients with distant metastases received RAI therapy (8 had metastases at diagnosis, while 8 developed distant disease during follow-up). Of these 16 patients, 11 exhibited iodine uptake and were subsequently treated with the radiopharmaceutical (17).

Zhang *et al*. (14) treated a patient with bilateral pulmonary metastases and multifocal liver metastases using a regimen of docetaxel and cisplatin, achieving complete resolution of all lesions. Similarly, F. Hameed *et al*. (18) described a patient with skull base and bone marrow metastases successfully treated with surgery, and Shrivastava *et al*. (19) reported a case of left mandibular body and ramus metastasis treated surgically and reconstructed with a microvascular osteomyocutaneous fibula flap (14).

In the present case, surgical resection of the suspected metastatic lesion was chosen as the radical treatment approach due to the lesion’s accessible location and the low associated morbidity. This strategy provided diagnostic confirmation and achieved complete biochemical remission. The patient remained disease-free during the 12-month follow-up period. A review of the English-language literature to date did not identify any previously reported cases of mandibular bone metastases from oncocytic thyroid carcinoma.

This article presents the second reported case of mandibular metastasis from oncocytic thyroid carcinoma and the first case of condylar metastasis.

## Conclusions

Oncocytic thyroid carcinoma is a more aggressive subtype with a poorer prognosis than other well-differentiated histological types due to its tendency to metastasize to regional lymph nodes and distant organs. It is estimated that approximately 5% of affected patients develop metastases. As a result, no standardized treatment protocol has been established for these lesions, with accepted therapeutic options including surgery, radioactive iodine (RAI), external beam radiation therapy, chemotherapy, and tyrosine kinase inhibitors.

In the present case, surgical resection of the right condylar metastasis was performed with curative intent, achieving complete excision and biochemical remission.

## Data Availability

The datasets used and/or analyzed during the current study are available from the corresponding author.
